# Supplementary material for: Potassium Dehydroandrograpolide Succinate Targets NRP1 Mediated VEGFR2/VE-Cadherin Signaling Pathway to Promote Endothelial Barrier Repair
Source: Int J Mol Sci. 2023 Feb 4;24(4):3096. doi: 10.3390/ijms24043096 (PMC9964616; doi:10.3390/ijms24043096)
Supplement: Supplementary file 1 [file ijms-24-03096-s001.zip › ijms-2143327-supplementary.pdf]

Supplementary

Potassium Dehydroandrograpolide Succinate Targets NRP1 Mediated VEGFR2/VE-Cadherin Signaling Pathway to Promote Endothelial Barrier Repair

Zheng Wang †, Xiao Wu †, Jiali Li, Qiru Guo, Zhong Jin, Hongfei Li, Bing Liang, Wangming Hu, Huan Xu, Liangqin Shi, Lan Yang and Yong Wang *

College of Basic Medicine, Chengdu University of Traditional Chinese Medicine, Chengdu, China
* Correspondence: wangyong@cdutcm.edu.cn
† These authors contributed equally to this work.

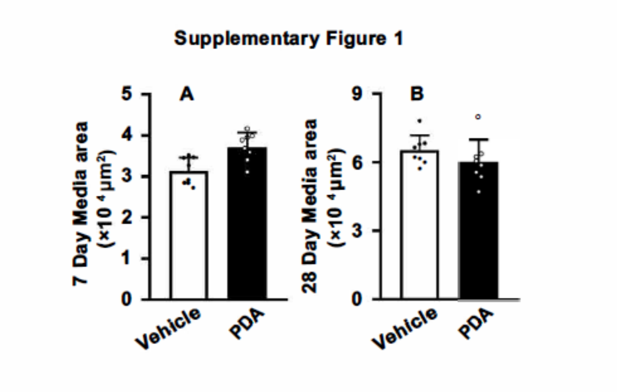

Figure S1. Analysis the media layer areas of carotid arteries after injury for 7 days and 28 days: (A) PDA (20 mg/kg) was administered by intraperitoneal injection for 7 consecutive days. The media layer area of the PDA group and the vehicle group was measured using Image J software (n = 8). (B) PDA (20 mg/kg) was administered by intraperitoneal injection for 28 consecutive days. The media layer area of the PDA and the vehicle group was measured using Image J software (n = 8). Data presented as mean ± SEM.

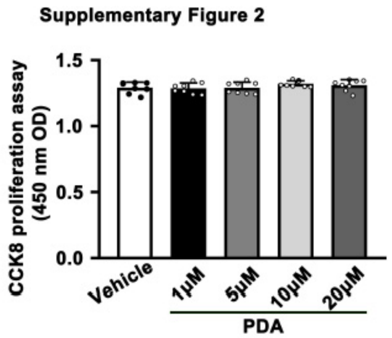

Figure S2. PDA treatment did not affect HUVEC-C viability: 1.5 × 10 3 HUVEC-Cs was seeded in 96-well culture plate, absorbance at 450 nm was evaluated using Cell Counting Kit-8 after PDA (1 μM, 5 μM, 10 μM, 20 μM) treatment for 12 hours (n = 8). Data presented as mean ± SEM. * P < 0.05.

Supplementary Figure 3

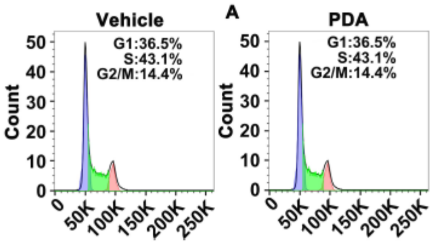

Figure S3. PDA regulates cell cycle: Treated HUVEC-Cs with PDA, and cell cycle was determined by flow cytometry after PI staining, 2×10^4 cells were sorted for each sample.

Supplementary Figure 4

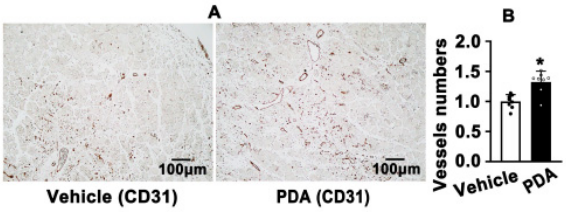

Figure S4. PDA promotes angiogenesis after skeletal muscle injury: (A) IF staining performed to evaluate expression of CD31 within the anterior tibial muscle of mice that were intramuscularly injected with cardiotoxin and treated with PDA (20 mg/kg) for 5 consecutive days, and the quantification data are shown in (B) (n = 8). Data presented as mean ± SEM. * P < 0.05.

Supplementary Figure 5

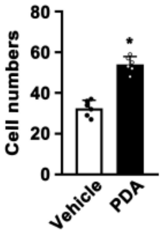

Figure S5. PDA promotes endothelial cell migration: The quantification data of the migrated cells in boyden chamber cell migration assay. Data presented as mean ± SEM. * P < 0.05.

Supplementary Figure 6

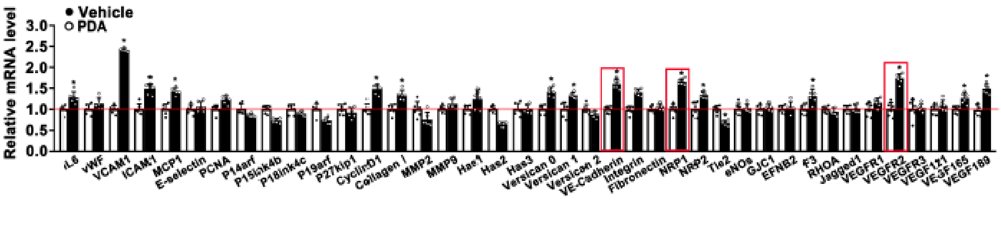

Figure S6. PDA regulates multiple signaling pathway: Treated the HUVEC-Cs with PDA (10 μM) for 24 hours, then the expression of relative mRNA level was detected by Real Time PCR (n = 6). Data presented as mean ± SEM. * P < 0.05.

Supplementary Figure 7

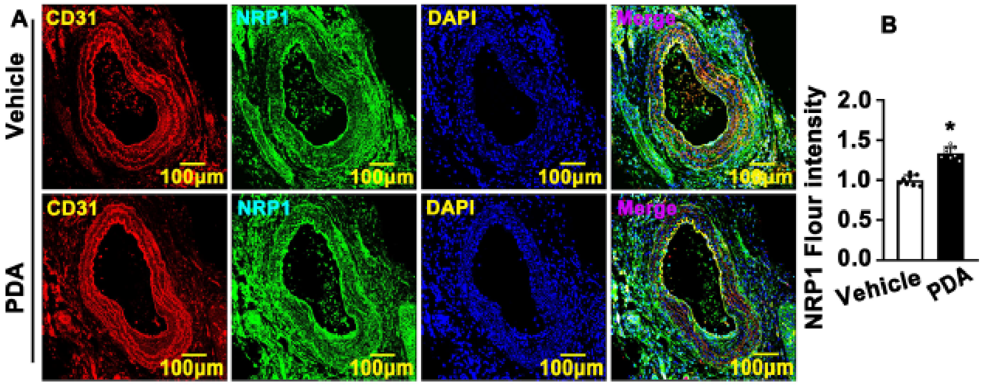

Figure S7. PDA promotes expression of NRP1 in carotid endothelial cells: (A) IF staining was performed against NRP1 antibody on the left carotid artery partial ligation model following PDA treatment for 28 days, and the quantification data was evaluated in (B) (n = 8). Data presented as mean ± SEM. * P < 0.05.

Supplementary Figure 8

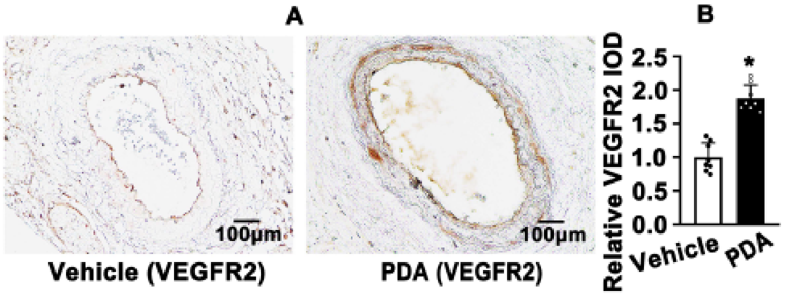

Figure S8. PDA promotes expression of VEGFR2 in carotid endothelial cells: (A) IF staining was performed to detect expression of VEGFR2 in the partially ligated carotid arteries. (B) The quantitative of VEGFR2 expression (n = 8). Data presented as mean ± SEM. * P < 0.05.

Supplementary Figure 9

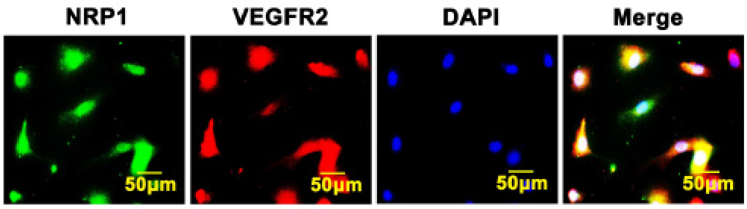

Figure S9. Co-expression of NRP1 and VEGFR2 in HUVECs: IF staining performed against NRP1 and VEGFR2 antibodies to determine whether co-localization expression of NRP1 and VEGFR2 in HUVECs.

Supplementary Figure 10

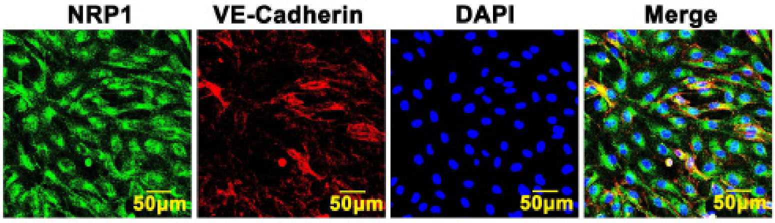

Figure S10. Co-expression of NRP1 and VE-Cad in HUVECs: IF staining performed against NRP1 and VE-Cad antibodies to determine whether co-localization expression of NRP1 and VE-Cad in HUVECs.

Supplementary Figure 11

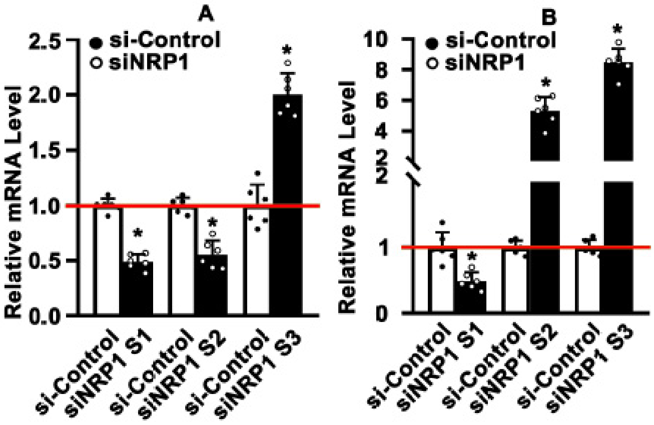

Figure S11. Knockdown of NRP1 in HUVEC and HUVEC-C by siRNA transfection: HUVECs (A) and HUVEC-Cs (B) were transfected with si-control or siRNA targeting NRP1, Real Time PCR performed to validate NRP1 deletion efficiency after transfection at 48 hours. S1 siRNA sequence was chosen for further studies. Data presented as mean ± SEM. * P < 0.05.

Supplementary Figure 12

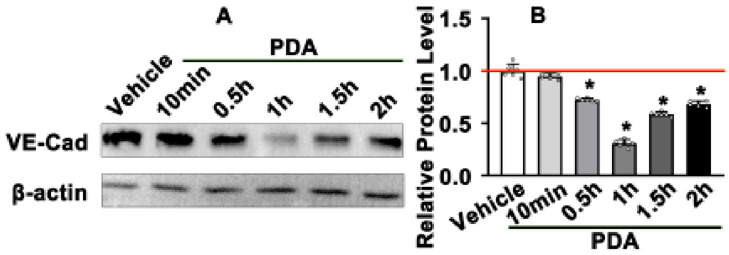

Figure S12. Dynamically expression of VE-Cad in HUVEC following PDA treatment: (A) Western blot was performed to determine the expression of VE-Cad in HUVECs after PDA (10 μM) treatment for different time points, and the quantification data are shown in (B) (n = 6). Data presented as mean ± SEM. * P < 0.05.

Table **S1. List of primer sequences used for Real Time PCR.**

Gene Name	*Species*	*Sequence*
IL-6	Human	F:5'-GGAGACTTGCCTGGTGAAAA-3'
IL-6	Human	R:5'-GTCAGGGGTGGTTATTGCAT-3'
vWF	Human	F:5'-GTCGAGCTGCACAGTGACATG-3'
vWF	Human	R:5'-GCACCATAAACGTTGACTTCCA-3'
VCAM1	Human	F:5'-TCAGATTGGAGACTCAGTCATGT-3'
VCAM1	Human	R:5'-ACTCCTCACCTTCCCGCTC-3'
ICAM1	Human	F:5'-GGCCGGCCAGCTTATACAC-3'
ICAM1	Human	R:5'-TAGACACTTGAGCTCGGGCA-3'
MCP1	Human	F:5'-TGCAGAGGCTCGCGAGCTA-3'
MCP1	Human	R:5'-CAGGTGGTCCATGGAATCCTGA-3'
E-selection	Human	F:5'-CCCGAAGGGTTTGGTGAG-3'
E-selection	Human	R:5'-TAAAGCCCTCATTGCATTGA-3'
PCNA	Human	F:5'-TCCGCCACCATGTTCGA-3'
PCNA	Human	R:5'-TATCCCAGCAGGCCTCGTT-3'
P14arf	Human	F:5'-ATGGTGCGCAGGTTCTTGG-3'
P14arf	Human	R:5'-TGCGGGCATGGTTACTGCCTC-3'
P15ink4b	Human	F:5'-CGCAGACCCTGCCACTCT-3'
P15ink4b	Human	R:5'-AGGCATCGCGCACGTC-3'
P18ink4c	Human	F:5'-CATCATGCAGCCTGGTTAGG-3'
P18ink4c	Human	R:5'-GCTGGCCGTGTGCTTCAC-3'
P19arf	Human	F:5'-CCCTCGTGCTGAGCTACTGA-3'
P19arf	Human	R:5'-ACCACCAGCGTGTCCAGGAA-3'
P27kip1	Human	F:5'-TGGAGAAGCACTGCAGAGAC-3'
P27kip1	Human	R:5'-GCGTGTCCTCAGAGTTAGCC-3'
CCND1	Human	F:5'-GTGCTGCGAAGTGGAAACC-3'
CCND1	Human	R:5'-ATCCAGGTGGCGACGATCT-3'
Collagen1	Human	F:5'-ATGCCTGGTGAACGTGGT-3'
Collagen1	Human	R:5'-AGGAGAGCCATCAGCACCT-3'
MMP-2	Human	F:5'-TGGCGATGGATACCCCTTT-3'
MMP-2	Human	R:5'-TTCTCCCAAGGTCCATAGCTCAT-3'
MMP-9	Human	F:5'-CCTGGGCAGATTCCAAACCT-3'
MMP-9	Human	R:5'-GCAAGTCTTCCGAGTAGTTTTGGAT-3'
Has1	Human	F:5'-GGAATAACCTCTTGCAGCAGTTTC-3'
Has1	Human	R:5'-GCCGGTCATCCCCAAAAG-3'
Has2	Human	F:5'-TCGCAACACGTAACGCAAT-3'
Has2	Human	R:5'-ACTTCTCTTTTTCCACCCCATTT-3'
Has3	Human	F:5'-AACAAGTACGACTCATGGATTTCCT-3'
Has3	Human	R:5'-GCCCGCTCCACGTTGA-3'
Versican 0	Human	F:5'-GACCTCAGGCGCTTTC-3'
Versican 0	Human	R:5'-CAGTGGTAACGAGATGCTTC-3'
Versican 1	Human	F:5'-GCGCCACCCTGTGAC-3'
Versican 1	Human	R:5'-CAGTGGTAACGAGATGCTTC-3'
Versican 2	Human	F:5'-GACCTCAGGCGCTTTC-3'
Versican 2	Human	R:5'-TAGCACTGCCCTTGGA-3'
VE-Cadherin	Human	F:5'-GAGCCGCCGCCGCAGGAAG-3'
VE-Cadherin	Human	R:5'-CGTGAGCATCCAGCAGTGGTAGC-3'
Integrin	Human	F:5'-TGGGCTACGGGCAGGAG-3'
Integrin	Human	R:5'-CAGCATTAACAGCAACAATCCG-3'
Fibronectin	Human	F:5'-CCGCCGAATGTAGGACAAGA-3'

Fibronectin	Human	R:5'-TGCCAACAGGATGACATGAAA-3'
NRP1	Human	F:5'-CAGAAAAGCCCACGGTCAT-3'
NRP1	Human	R:5'-CAGCCAAATTCACAGTTAAAACC-3'
NRP2	Human	F:5'-AAGTCTCCTACAGCCTAAACGG-3'
NRP2	Human	R:5'-GATGTCAGGGGTGTCATAGTGC-3'
Tie2	Human	F:5'-TTGAAGTGGAGAGAAGGTCTG-3'
Tie2	Human	R:5'-GTTGACTCTAGCTCGGACCAC-3'
eNOs	Human	F:5'-TGATGGCGAAGCGAGTGAAG-3'
eNOs	Human	R:5'-ACTCATCCATACACAGGACCC-3'
GJC1	Human	F:5'-AGCTGTAGGAAGGAGAATCCATC-3'
GJC1	Human	R:5'-TGCAAACGCATCATAACAGACA-3'
EFNB2	Human	F:5'-TTCGACAACAAGTCCCTTTG-3'
EFNB2	Human	R:5'-GATGTTGTTCCCCGAATGTC-3'
F3	Human	F:5'-CCACTACAAATACTGTGGCAG-3'
F3	Human	R:5'-TCCAATCTCCTGACTTAGTG-3'
RHOA	Human	F:5'-GAAGAGGCTGGACTCGGATT-3'
RHOA	Human	R:5'-AGCAAGCATGTCTTTCCACA-3'
Jagged1	Human	F:5'-CAGGACCTGGTTAACGGATTT-3'
Jagged1	Human	R:5'-GCCTCACATTTGCATC-3'
VEGFR1	Human	F:5'-TCTCACACATCGACAAACCAATACA-3'
VEGFR1	Human	R:5'-GGTAGCAGTACAATTGAGGACAAGA-3'
VEGFR2	Human	F:5'-GCAGGGGACAGAGGGACTTG-3'
VEGFR2	Human	R:5'-GAGGCCATCGCTGCACTCA-3'
VEGFR3	Human	F:5'-GACAGCTACAAGTACGAGCATCTG-3'
VEGFR3	Human	R:5'-CGTTCTTGCAGTCGAGCAGAA-3'
VEGF121	Human	F:5'-CCCTGATGAGATCGAGTACATCTT-3'
VEGF121	Human	R:5'-GCCTCGGCTTGTCACATTTT-3'
VEGF165	Human	F:5'-CCCTGATGAGATCGAGTACATCTT-3'
VEGF165	Human	R:5'-AGCAAGGCCCACAGGGATTT-3'
VEGF189	Human	F:5'-CCCTGATGAGATCGAGTACATCTT-3'
VEGF189	Human	R:5'-AACGCTCCAGGACTTATACCG-3'

Table S2. The sequences used for siRNA.

Gene Name	*Species*	*Sequence*
NRP1-Homo-476	Human	S:5'-CAGAGAAUUAUGAUCAACUTT-3'
NRP1-Homo-476	Human	AS:5'-AGUUGAUCAUAAUUCUCUGTT-3'
